# Intelligence Sensors and Sensing Spaces for Smart Home and Environment

**DOI:** 10.3390/s22082898

**Published:** 2022-04-09

**Authors:** Mi Jeong Kim, Han Jong Jun

**Affiliations:** School of Architecture, Hanyang University, Seoul 04763, Korea

## 1. Introduction

Practitioners in the domains of architecture, engineering, and construction have conducted considerable research on smart homes and smart environments. Many studies on smart homes and environments have dealt with monitoring residents’ behaviors by sensors and supporting health through smart services to enable a comfortable lifestyle for and the independence of residents. By embedding intelligent sensors in the built environment, our living spaces have been digitalized into interactive and smart sensing spaces, affecting residents’ experiences. We are currently facing big challenges due to the COVID-19 pandemic, which has caused dramatic changes in our daily life. Many experts predict that overall lifestyle and spatial experience will be changed, calling for new working and living styles and more sustainable environments. Smart homes and environments equipped with sensors could be at the center of urban activities. Sensing spaces comprise a critical research area for moving towards the realization of responsive and intelligent environments from a cognitive perspective. This Special Issue identifies and introduces state-of-the-art developments focusing on sensor technologies and sensing spaces, contributing to smart homes and environments. The advancement in information and communication technologies (ICTs), the Internet of Things (IoT), machine learning (ML), and artificial intelligence (AI) have enabled the implementation of smart systems and interactive environments. Significant changes caused by such advanced technologies in built environments have affected occupants’ perception, cognition, and experience of their spaces. Thus, considerable research on sensing spaces has been performed from user and cognitive perspectives. Ten high-quality papers were published in this Special Issue. Two papers are systematic review articles, and eight papers are original research articles. Among the eight research articles, four papers are associated with smart homes, innovative campuses, and intelligent station spaces. The other four papers deal with methodological approaches in original research and experimental works.

## 2. Overview of Contribution

A digital ecosystem is a large-scale, ubiquitous system, facilitated by advanced ICTs and digital actors—the interactive activities of which are sustainably evolving. The authors of [1] systematically characterize smart environments as interactive and collective platforms in a digital ecosystem by reviewing scholarly sources to understand intelligent environments from an architectural perspective. The behaviors and characteristics of responsive architecture were reexamined, where the term “responsive” refers to either adaptive or intelligent activities. Based on the systematic review, the way in which sensors and sensing information are designed to build smart environments is proposed.

Combining generative design (GD) with building information modelling (BIM) provides an intelligent design approach and generates construction information. The authors of [2] performed a critical review of approaches to developing GD-BIM, allowing the analysis of the methodological relationships, skill requirements, and improvement strategies of GD-BIM systems. Perspectives of objective-oriented, GD-component-based, and skill-driven GD-BIM development, as well as reference guides, were proposed. Designers in the building industry can select appropriate methods and formulate skill-improving paths to develop GD-BIM systems based on the information presented by the review.

An integrated smart home system (ISHS) has the potential to improve quality of life for the elderly population; however, their willingness to adopt an ISHS is essential for this development. The authors of [3] conducted an empirical study to investigate the perceptions of the elderly population on ISHSs through technical trials followed by focus group interviews based on four factors: perceived comfort, perceived usability, perceived privacy, and perceived benefit. The results indicate that elderly participants gave negative responses regarding usability, complexity, and discomfort with daily activities. Contrary to expectations, the elderly participants agreed to share monitoring information with potentially helpful entities, due to their acknowledgement of their frailty of old age; in sharing this information, they endorsed the benefits of ISHS.

Biophilic design integrates nature into architecture to improve health and wellbeing, and smart home services (SHSs) enhance the convenience of household activities through ensuring the independence of the elderly population in their homes. The authors of [4] surveyed a biophilic-experience-based SHS to identify the elderly population’s preferred services, and discussed the configuration of the sensors and devices for the implementation of the services. The results of the study show that the specified services were mainly attributed to “immersion and interaction with nature”, “management of wellbeing and indoor environmental quality”, and “natural process and systems”.

In the current environmental and climatic context, the authors of [5] proposed a novel multidisciplinary approach to the campus environment, namely a “smart tree”, where architecture, greening, and ICT systems combine to provide a sustainable sensing area for leisure study and co-working. In a small, innovative outdoor area, a prototype of a “smart tree” was implemented to test the proposed concepts—“environmental comfort” and “circular architecture”—of the smart campus as an element of the smart city paradigm. In the context of the COVID-19 pandemic, the “smart tree” would provide a seasonal alternative to working inside the campus buildings, with the added safety of the open air.

Existing closed subway stations are crucial spaces that need to be renovated into pleasant environments, because many citizens use them daily. Based on the assumption that green elements would enhance the physical and psychological health of the population in the indoor environment, the authors of [6] identified strategies for implementing a pleasant and healthy indoor landscape in a subway station space. Eight 3D landscape models of the subway interior were developed, and the psychophysical responses of 60 participants were investigated based on their greenness index preference. The results showed that the participants preferred the model with a greenness index of 15% for underground subway stations, whereas they preferred the model with a greenness index of 10% for aboveground subway stations.

In emergencies, people’s evacuation behavior manifests in crowd behavior, causing chaos and accidents, and drills are a vital activity in increasing the preparedness of a group to respond safely in emergencies. Focusing on exit familiarity, visibility, and panic effects, the authors of [7] conducted simulation experiments in GIS and multi-agent systems by testing different exit familiarity ratios to identify the emergency response patterns of drill-trained individuals. The results showed that evacuation training level affected the evacuees’ choice of exit, thus affecting the escape process. The optimized evacuation plan presented in the study could improve the overall efficiency of evacuation through the self-organization effect.

Wayfinding is associated with a series of cognitive processes, such as understanding the surrounding environment, establishing a plan, converting it into a behavioral activity, and making a decision at each point. The authors of [8] conducted experiments at a train station and investigated participants’ emotional perception of space and type of movement patterns depending on their spatial familiarity; in the experiment, the authors extracted influence factors for wayfinding. The result showed that the group which was familiar with the space complained of more discomfort, recognizing these problems more than the other group, since their spatial familiarity made them biased towards negative factors.

Collecting meaningful data to optimize urban resources is critical for the implementation of smart cities; such data contribute to intelligent street management and neighborhood planning strategies. The authors of [9] developed an automated detection method to obtain sidewalk GIS data, using computer vision and ML with Google Street View data. The results show that the image-level and the street-level sidewalk classifiers performed with nearly 90% accuracy in most cases.

The adoption of ML to detect specific cognitive symptoms have been used extensively in social media contexts; however, the application of ML in the diagnosis and treatment of mental illness is yet undeveloped. The authors of [10] examined whether ML could effectively detect signs of schizophrenia in social media users and established coherent semantic groups of words which are key to detecting schizophrenia, including symptom-related words, negative sentiment words, and words related to mental health support.

## 3. Conclusions

The two review papers presented in this Special Issue have significance through their provision of a solid knowledge base for understanding and developing sensing spaces by characterizing smart environments and GD-BIM systems. To ensure a positive influence on occupants’ daily living, target user groups’ experiences were examined in indoor spaces, intelligent services, and outdoor areas, focusing on adopting technologies, biophilic designs, and multidisciplinary approaches, respectively. Furthermore, intelligent environments were implemented to simulate emergency situations under different conditions to examine occupants’ perceptions and behaviors depending on the given variables. Understanding how people perceive and behave in a specific environment or situation is critical knowledge for the prevention of uncomfortable experiences and dangerous situations in a built environment. Obtaining meaningful data to solve current problems is crucial for realizing intelligent environments and innovative systems. Researchers have adopted ML to detect important information related to their field, including public sidewalk GIS data and the linguistic characteristics of schizophrenia. Through a continuation of the research efforts presented in this Special Issue, we can accomplish advanced environments and systems to support the positive experiences and healthy lifestyles of the population.
